# The Association Between Depression and Antidiabetic Treatments in Type 2 Diabetes Patients with Both Good and Poor Glycemic Control

**DOI:** 10.3390/jcm14103460

**Published:** 2025-05-15

**Authors:** Perihan Ozkan Gumuskaya, Ozgur Altun, Emine Yildirim, Nur Karakutuk Yuztas, Neslihan Ozsoy, Semih Kalyon, Leyla Irak, Mustafa Ozcan, Zeren Ozturk Altun, Pınar Saner Demir, Eylem Ozgun Cil, Yucel Arman, Hafize Uzun, Tufan Tukek

**Affiliations:** 1 Department of İnternal Medicine, Prof. Dr. Cemil Tascioglu City Hospital, University of Health Sciences, Istanbul 34668, Turkey; ozgurakademik@gmail.com (O.A.); nurkarakutuk@hotmail.com (N.K.Y.); nes_ozsoy@yahoo.com.tr (N.O.); semihkalyon@hotmail.com (S.K.); mozcandr@hotmailz.com (M.O.); drpinardemir81@gmail.com (P.S.D.); dreylemozgun@hotmail.com (E.O.C.); dryarman@yahoo.com (Y.A.); 2 Department of General Surgery, Faculty of Medicine, Istanbul Atlas University, Istanbul 34403, Turkey; opdreyildirim@gmail.com; 3 Department of Endocrinology and Metabolism, Prof. Dr. Cemil Tascioglu City Hospital, University of Health Sciences, Istanbul 34668, Turkey; leylairak@hotmail.com; 4 Department of Psychiatry, Prof. Dr. Cemil Tascioglu City Hospital, University of Health Science, Istanbul 34668, Turkey; zerenoz@yahoo.com; 5 Department of Medical Biochemistry, Faculty of Medicine, Istanbul Atlas University, Istanbul 34403, Turkey; huzun59@hotmail.com; 6 Department of Internal Medicine, Istanbul Faculty of Medicine, Istanbul University, Istanbul 34452, Turkey; tufantukek@hotmail.com

**Keywords:** depression, diabetes, glycemic control, HbA1c, antidiabetic treatments

## Abstract

**Background**: Much research has demonstrated that there is a relation between depression and chronic diseases. Type 2 diabetes mellitus (T2DM) is one of the most common chronic diseases, and it is an expanding global health problem. This study aims to explore the relationship between glycemic control (GC), antidiabetic agents, and depression in T2DM, focusing on how depression affects treatment adherence and GC. **Methods**: This prospective study included 250 patients with T2DM. Demographic information and laboratory results were obtained from the patients as well as from the hospital’s laboratory system. Beck’s Depression Inventory (BDI) scores, GC indicator HbA1c levels, and antidiabetic agents used by the patients were compared. **Results**: Upon analyzing the findings, we found a statistically significant positive correlation between the severity of depression and HbA1c levels (*p* < 0.001). Additionally, no significant relationship was observed between depression and the use of oral antidiabetic medications, except for metformin. However, a significant association between depression and insulin use was found even in patients with good GC (*p* < 0.001), regardless of whether HbA1c was high. **Conclusions**: Regardless of the type of antidiabetic treatment, diabetic patients receiving intensive insulin therapy, whether they have good or poor GC, should be carefully evaluated for depressive symptoms. Appropriate psychiatric support should be provided to help achieve GC and enhance their quality of life. Our study is the first to emphasize the importance of close monitoring for diabetic patients using insulin, even those with good GC. Understanding these interactions may improve disease management, patient outcomes, and quality of life.

## 1. Introduction

Type 2 diabetes mellitus (T2DM) is one of the most common chronic diseases. Recent studies indicate that individuals with DM are at a higher risk of developing depression. The prevalence of depression is nearly twice in individuals with T2DM [1]. Diabetes and depression may share a common etiology, with diabetes potentially increasing the risk of developing depression, and depression, in turn, heightening the risk of diabetes [2,3]. Several mechanisms have been proposed to explain the comorbidity between depression and T2DM, including stress and inflammation, which have been explored in numerous studies [2].

Depression can manifest in different ways, and its effects can vary in intensity, but these disruptions in emotions, cognition, and behaviors can make daily life challenging [2,4]. It decreases the quality of life [2,5]. Chronic stress causes chronic hypercortisolemia, insulin resistance (IR), obesity, and T2DM; also, cortisol may activate the anxiety state, producing depression [2,6]. Inflammatory cytokine levels increase during chronic stress because of immune dysfunction [2]. These cytokines disturb the pancreatic beta cell function, causing diabetes, and disturb the neurotransmitters, causing depression [2,7]. The presence of depression in individuals with diabetes can worsen the overall prognosis of the condition. It may lead to non-compliance with treatment [2,8]. The various pathogenic factors involved in T2DM necessitate the use of multiple antidiabetic medications to achieve and maintain normoglycemia. Treatment must be not only effective and safe but also enhance the patient’s quality of life [9].

Glycemic control (GC), T2DM, antidiabetic agents, and depression are all interrelated factors that significantly influence the management and progression of diabetes. Proper GC is crucial for managing T2DM, and the choice of antidiabetic agents plays a vital role in achieving this. However, depression, which is common in individuals with T2DM, can negatively impact adherence to treatment, thereby complicating the overall management of the condition [10]. Understanding the interplay between these factors is essential for improving patient outcomes and quality of life.

Despite its significance, adherence to GC has been found to be low due to a variety of factors. Identifying the factors that affect GC is crucial for initiating appropriate interventions to improve GC. There is limited information regarding the role of depression in uncontrolled T2DM. The aim of this study was to investigate the interrelationship between GC, antidiabetic agents, and depression in T2DM. Specifically, it seeks to explore how depression affects treatment adherence and GC in individuals with T2DM, and how these factors influence the management and progression of the disease. By understanding the complex interaction between these variables, the study aims to improve patient outcomes and quality of life.

## 2. Materials and Methods

### 2.1. Ethical Approval

All procedures performed in the study involving human participants were in accordance with the ethical standards of the institutional and/or national research committee and with the 1964 Helsinki declaration and its later amendments or comparable ethical standards. Approval for this study was obtained from Prof. Dr. Cemil Tascioglu City Hospital Ethics Committee for Clinical Studies in February 2020 (18 February 2020; Decision no 65; Number: 48670771-514.10).

### 2.2. Subjects

The research was designed as a prospective study and included 250 patients diagnosed with T2DM and evaluated during routine outpatient check-ups in Prof. Dr. Cemil Tascioglu City Hospital between February 2020 and April 2020. All these 250 patients met the study criteria.

### 2.3. The Inclusion Criteria

(i) Having given consent to participate in the study; (ii) Not having any other chronic diseases other than T2DM; (iii) To be over 18 years old and less than 80 years old; (iv) Not taking antidepressants; (v) Have not had a recent event that could cause depression; (vi) At least 1 year since T2DM diagnosis; (vii) Stable medications within 4 weeks before screening.

### 2.4. The Exclusion Criteria

We did not include the patients with a known history of diabetic complications. Based on a detailed physical examination and detailed anamnesis information recorded in our country’s health registration system (e-pulse system), patients with diabetic retinopathy, impaired vision, previous myocardial infarction, heart failure, diabetic foot, peripheral artery disease, extremity amputation due to diabetes, chronic renal failure, previous cerebrovascular accident, and transient ischemic attack history or symptoms were not included in this study.

(i) Refuse to participate in the study; (ii) Patients with chronic diseases other than T2DM; (iii) Patients taking antidepressant therapy or have had a recent event that could cause depression; (iv) Pregnancy or nursing; (v) People lacking of self-care and having a sedentary life; (vi) Patients with lipohypertrophy because of insulin treatment; (vii) Patients having diabetic polyneuropathy; (viii) Patients with low socioeconomic status, unfavorable and stressful life style; (ix) Patients with substance abuse; (x) Patients with frequent hypoglycemic symptoms.

DM was defined as per the American Diabetes Association (ADA) criteria [11]. Data were retrieved from each patient with a diagnosis of T2DM with respect to medical history, age and sex, the laboratory data including glycated hemoglobin (HbA1c), and 2-h postprandial glucose level, and antidiabetic therapeutic agents. The diabetes status was assessed in the short-term clinical control by HbA1c, as *mild* if HbA1c < 7, *moderate* if HbA1c: 7–9, and *severe* if HbA1c > 9. HbA1c < 7.0% and HbA1c > 7.0% were defined as good GC and poor GC, respectively.

### 2.5. Patients with T2DM Were Classified Based on the Beck Depression Inventory (BDI) Scores

All patients were questioned about previous medical diagnosis of depression and if any, removed from the study. Each of the symptoms in the BDI fit the diagnostic criteria of the DSM-IV, and the total time it should take a person to complete the questionnaire is five minutes. The patients were classified according to BDI as normal (Beck score 0–9) or having mild (Beck score 10–16), moderate (Beck score 17–29), and severe (Beck score 30–63) depression [12].

### 2.6. Statistical Analysis

Data obtained in our study were analyzed statistically with the SPSS 21 software program. Normality control of continuous variables was evaluated with the Shapiro–Wilk test. One-way ANOVA was used in the comparisons according to Beck classification for the variables conforming to the normal distribution, and Tukey was used as one of the post hoc tests. The Kruskal–Wallis test was used for those who did not comply with the normal distribution. The chi-square test was used in the analysis of categorical data.

## 3. Results

We found that the mean age varied across the BDI scale (BDIS) groups (*p* = 0.003). Accordingly, the mean age of the group with severe depression was higher than the normal group (*p* < 0.05). Body mass index (BMI) medians differed according to Beck groups (*p =* 0.029). The median BMI was higher in the severe depression group than in the normal group (*p* < 0.05). The median duration of diabetes was found to differ between the BDI groups (*p* < 0.001). We found that the median duration of diabetes was higher in the moderate and severe groups than in the normal group (*p* < 0.05). We observed significant differences in gender ratios across the BDI groups (*p* < 0.001). The prevalence of mild, moderate, and severe depression was found to be higher in women compared to those with normal depression levels (*p* < 0.05) (Table 1).

There was a significant difference in median HbA1c levels between BDI groups (*p* < 0.001). The median HbA1c was higher in the moderate and severe depression groups compared to the mild and normal groups (*p* < 0.05). As another result, fasting blood glucose (FBG) medians differed according to BDI groups (*p* < 0.001). The median FBG of the normal group was lower than that of the moderate and severe group, and the median FBG of the mild group was lower than that of the severe group (*p* < 0.05). Also, postprandial blood glucose (PBG) medians differed according to Beck depression groups (*p* < 0.001). The median PBG was higher in the moderate and severe groups than in the normal and mild groups (*p* < 0.05) (Table 2). A statistically significant relationship was found between BDI and HbA1c levels (*p* < 0.001).

According to this, while the values of HbA1c were less than 7, the rate of moderate and severe depression was lower than the normal and mild groups. On the other hand, when HbA1c values were greater than 9, the rate of moderate and severe depression was higher compared to the normal and mild groups (*p* < 0.05) (Table 3).

We found a statistically significant relationship between depression and metformin (*p* = 0.015). The proportion of patients using metformin in the moderate depression group was lower compared to the normal and mild depression groups (*p* < 0.05). Another finding was a statistically significant relationship between BDIS and the use of mixed insulin (*p* = 0.005). It was seen that the mixed insulin ratio of groups with moderate and severe depression was higher than the groups with normal or mild depression (*p* < 0.05). Also, a statistically significant relationship was found between Beck depression groups and intensive insulin treatment (basal plus bolus insulin treatment) (*p* = 0.005). The proportion of patients receiving basal plus bolus insulin treatment was lower in the normal, mild, and moderate depression groups compared to the severe depression group. Additionally, the basal plus bolus insulin ratio in the normal and mild depression groups was lower than that in the moderate depression group (*p* < 0.05) (Table 4).

According to logistic linear regression analysis, various factors that have a significant effect on depression scores were determined (Table 5). It was observed that female gender significantly increased depression scores (B = 4.14; *p* < 0.001). In addition, an increase in depression scores was observed with increasing age (B = 0.24; *p* < 0.001). Postprandial blood sugar values also positively predicted depression scores, and this relationship was found to be statistically significant (B = 0.02; *p* = 0.014). As BMI increased, an increase in depression scores was also observed, and this relationship was found to be significant (B = 0.23; *p* = 0.013). Although a positive relationship was found between triglyceride levels and depression, this finding did not reach the borderline statistical significance level (B = 0.01, *p* = 0.085). Insulin treatment types were also among the important variables affecting depression scores. Depression scores were found to be significantly higher in individuals using mixed insulin (B = 5.27, *p* < 0.001). Similarly, depression levels were found to be significantly higher in individuals using basal insulin (B = 5.85; *p* < 0.001) and intensive insulin therapy (B = 8.73; *p* < 0.001).

According to multiple linear regression analysis, various variables were determined that significantly predicted depression scores (Table 6). Female gender was found to be associated with a significant increase in depression scores (B = 4.23, *p* < 0.001). Similarly, it was observed that depression scores increased with increasing age (B = 0.23, *p* < 0.001). Body mass index (BMI) was also found to have a positive and significant effect on depression scores (B = 0.23, *p* = 0.014). When evaluated in terms of insulin treatment types, it was determined that individuals who received mixed insulin (B = 5.08, *p* < 0.001), basal insulin (B = 5.44, *p* < 0.001), and intensive insulin treatment (B = 7.95, *p* < 0.001) had significantly higher depression scores. These findings show that there is a strong relationship between the type of insulin used and the level of depression. In addition, it was observed that HbA1c level was one of the variables with the highest standardized effect on depression scores (β = 0.37), and this relationship was found to be statistically highly significant (B = 1.50, *p* < 0.001).

## 4. Discussion

The most important finding of the present study is the significant positive correlation between the severity of depression and HbA1c levels, indicating that higher depression severity is associated with poor GC. While no significant relationship was found between depression and the use of oral antidiabetic medications, metformin usage was an exception. Additionally, depression was significantly associated with insulin use, even among patients with good GC, regardless of HbA1c levels. Our findings suggest a notable association between insulin use and depression, even among patients with apparently good glycemic control. However, we acknowledge that we did not present detailed stratified data showing HbA1c values across the spectrum of depression severity. While previous studies have reported a higher prevalence of insulin therapy in patients with moderate to severe depression, our data do not allow us to definitively conclude whether this relationship is independent of HbA1c levels. This highlights the need for further research using more granular data to explore this association.

The results of the present study indicate several noteworthy findings. The higher median BMI in the severe depression group compared to the normal group suggests that depression may be associated with an increased risk of obesity or weight gain, which can complicate diabetes management. The significant difference in the median duration of diabetes across the BDI groups further highlights that depression may affect long-term disease progression, with the moderate and severe depression groups having a longer duration of diabetes than the normal group. Additionally, the observed gender differences in depression prevalence, with higher rates of mild, moderate, and severe depression in women, align with the existing literature, indicating that women may be more susceptible to depression in the context of chronic conditions like diabetes. Women with diabetes experience a higher prevalence of depression compared to men [1,13]. Similarly, in our study, the rates of mild, moderate, and severe depression in women were found to be higher than those in the normal depression group. According to Albei et al. [14], patients with T2DM have an increased rate of anxiety and depression due to persistent hyperglycemia and aging, which is expressed in a lower quality of life. These findings underline the importance of considering gender and mental health status in the management of diabetes, as both factors appear to play a critical role in disease progression and management outcomes.

The results of the present study reveal a strong association between depression severity and GC markers. The significant difference in median HbA1c levels between the BDI groups, with higher HbA1c observed in the moderate and severe depression groups compared to the mild and normal groups, suggests that depression may contribute to poor GC. This finding is consistent with previous research that links mental health status to diabetes management, and higher depressive symptoms may affect GC in patients with T2DM [15,16,17,18]. Additionally, the differences in median FBG and PBG levels across the BDI groups further emphasize the impact of depression on GC. Specifically, the normal group had lower median FBG levels than both the moderate and severe depression groups, and the mild group had lower FBG than the severe group. Similarly, the median PBG was significantly higher in the moderate and severe depression groups than in the normal and mild groups. These findings highlight the potential role of depression in exacerbating blood glucose levels, making it essential to address both GC and mental health when managing diabetes. The statistically significant relationship between BDI scores and HbA1c levels underscores the importance of considering depression as a factor that may hinder effective diabetes management and improve patient outcomes.

According to the literature, there is limited evidence suggesting that depression could be a potential side effect of insulin, biguanides, and/or sulfonylureas [19]. Berge et al. [20] noted that the use of insulin and sulfonylureas is associated with weight gain, and this weight gain or obesity can contribute to increased stress. In the study by Papelbaum et al. [21], comorbid depression in diabetes was suggested as one of the potential factors contributing to inadequate GC. Their purpose was to investigate the association between major depression and the GC of T2DM. The authors explore the clinical relevance of these findings in the standard treatment of diabetes. In the current study, in addition to this goal, we also aimed to examine the relationship between depression and antidiabetic treatment agents. The results indicate several important associations between depression and antidiabetic treatments. A statistically significant relationship was found between depression and metformin use, with the moderate depression group having a lower proportion of patients using metformin compared to the normal and mild depression groups. However, considering this result and our current patient number, we thought that we could not make a definitive judgment about metformin. Additionally, a significant relationship was observed between BDIS and the use of mixed insulin. The use of mixed insulin was more prevalent in the moderate and severe depression groups than in the normal or mild depression groups, highlighting that depression might contribute to more complex insulin regimens. Furthermore, a significant relationship was found between depression severity and intensive insulin treatment (basal plus bolus insulin therapy). The proportion of patients receiving basal plus bolus insulin was lower in the normal, mild, and moderate depression groups compared to the severe depression group. Notably, the basal plus bolus insulin ratio was lower in the normal and mild depression groups compared to the moderate depression group. These findings suggest that severe depression may lead to more intensive insulin regimens, possibly due to poor GC associated with higher depression severity. Overall, these results underline the importance of considering depression when selecting antidiabetic treatments, as it appears to influence both medication choices and insulin therapy intensity.

Anderson et al. [22] conducted a meta-analysis of 42 studies examining the relationship between depression and diabetes. The authors found that the presence of diabetes doubles the likelihood of developing depression. They did not mention the relation with antidiabetic agents. The findings of Min Guo et al. [23] suggested that chronic treatment with metformin may have antidepressant-like behavioral effects. Kessing et al. [24] demonstrated that continued use of metformin, as well as combinations of drugs including metformin, were associated with lower rates of depression. Similarly, Perry et al. [25] found a positive association between IR and depression. Metformin remains the most widely prescribed insulin-sensitizing agent in clinical practice [26]. Interestingly, in our study, a statistically significant relationship was found between depression and metformin use in the mild and moderate depression groups. However, no statistically significant relationship was observed between depression and metformin combination therapies. Additionally, Berge et al. [27] found no association between depression and insulin use. The four antidiabetic agents tested—vildagliptin, glyburide, pioglitazone, and metformin—demonstrated antidepressant effects in diabetes- and reserpine-induced depression models. The differences in their effectiveness in DM rats may be attributed to factors such as their ability to reach the central nervous system, their antioxidant and anti-inflammatory properties, and their capacity to reduce the activation of the hypothalamic–pituitary–adrenal (HPA) axis. Moreover, these findings suggest promising potential for further research into the use of antidiabetic drugs alone as possible alternatives to traditional antidepressant medications in the treatment of diabetes-associated depression [28]. The results highlight the potential for further research into antidiabetic drugs as possible alternatives to conventional antidepressants, especially in the context of diabetes-associated depression. These findings open up new avenues for developing treatment strategies that could address both metabolic and mental health concerns in diabetic patients.

Recent trials have shown connections between insulin signaling and depression. Knockdown of insulin receptors in the hypothalamus or astrocytes has been linked to depressive behavior in mice, and insulin administration has been shown to influence mood and cognition in several human studies [29,30,31,32,33,34]. Intranasal delivery of insulin in awake mice can facilitate the passage of regulatory and metabolic hormones across the blood–brain barrier, resulting in improved mood and reduced anxiety in rats [35,36]. The results of studies investigating the relationship between insulin treatment and depression are controversial. Bell et al. [37] reported a negative correlation between depression and insulin treatment, while Noh et al. [38] and Al-Amer et al. [39] found a positive correlation between insulin treatment and depression. Bai et al. [40] demonstrated that patients on insulin therapy had lower endogenous insulin levels, making them more susceptible to metabolic dysregulation. They confirmed that insulin therapy was significantly associated with an increased risk of depressive symptoms [40]. In our study, similarly to other studies, we also found a statistically significant relationship between diabetes and depression. However, our unique contribution is the emphasis on the statistically significant relationship between insulin use and depression in patients with good GC, a topic that has not been explored in previous research. Based on these findings, the success of any treatment approach for T2DM patients is largely contingent on providing psychological support and implementing preventive strategies that promote and sustain mental well-being [41]. When the model is evaluated in general, it is seen that both demographic and metabolic parameters make significant contributions to depression scores; especially female gender, age, BMI, postprandial blood sugar, and the types of insulin treatment applied are important predictors. This finding indicates that the level of glycemic control may be closely related to psychological status. In general, the model reveals that both demographic (gender, age, BMI) and clinical (type of insulin treatment, HbA1c) variables have significant effects on depression. HbA1c and intensive insulin treatment were among the strongest predictors. Although HbA1c was used as an indicator of long-term glycemic control in the current study, it does not distinguish between fasting and postprandial hyperglycemia. Additionally, we did not assess insulin-like growth factor (IGF) levels or glycemic variability, which could have an independent impact on depression, even in patients with normal HbA1c levels. Future research using continuous glucose monitoring or investigating IGF levels may provide a clearer understanding of these relationships.

### Limitations of the Study

This study has several limitations. First, it was conducted in a single center with a relatively small sample size (n = 250), which may limit the generalizability of the findings. Second, the variation in antidiabetic medications used by participants could influence both glycemic control and depression levels, and due to the sample size, subgroup analyses for all treatment types were limited. Third, the study period coincided with the early phase of the COVID-19 pandemic (February–April 2020), which may have independently influenced depressive symptoms due to increased emotional stress, anxiety, and uncertainty during that time. In addition, although we observed a significant relationship between insulin use and depression among patients with good glycemic control, we were not able to present stratified data by HbA1c levels across different depression severity categories. Therefore, our interpretation of the relationship between insulin use and depression, independent of glycemic control, should be considered with caution. These factors should be considered when interpreting the results. Future multicenter studies with larger populations and consideration of psychosocial influences are warranted.

## 5. Conclusions

Our study supports previous research that has shown inadequate GC in the presence of both depression and T2DM. We demonstrated that diabetic patients with high HbA1c levels, irrespective of their antidiabetic treatment, and those on intensive insulin therapy despite having normal HbA1c levels, should be closely monitored for depressive symptoms. HbA1c and intensive insulin treatment were the strongest predictors in the presence of both depression and T2DM. Additionally, HbA1c levels should be managed, and their quality of life should be enhanced by providing necessary psychiatric support. Our study is the first to highlight the importance of closely monitoring insulin-treated diabetic patients, even those with good GC, for potential depressive symptoms.

## Figures and Tables

**Table 1:** Beck score evaluations according to age, BMI, duration of disease, and gender.

	Beck Score								Total		
	Normal		Mild		Moderate		Severe				
	Mean ± SD	Median (IQR)	Mean ± SD	Median (IQR)	Mean ± SD	Median (IQR)	Mean ± SD	Median (IQR)	Mean ± SD	Median (IQR)	*p*1
**Age (year)**	57.29 ± 8.7	58 (51–62)	60.65 ± 10.14	61 (51–67)	61.54 ± 10.72	60.5 (54.75–68)	66.33 ± 11.22 ^a^	71 (54.25–73)	60.66 ± 10.36	61 (52.75–68)	0.003
**BMI (kg/m^2^)**	28.89 ± 3.97	27.8 (26.1–31.1)	29.93 ± 4.29	29.87 (27.05–32.1)	30.02 ± 4.55	29.05 (26.5–32.28)	31.57 ± 4.49	31.3 (29.35–34.45) ^a^	29.87 ± 4.35	29.4 (26.7–32)	0.029 *
**Duration of diabetes (year)**	7.17 ± 5.09	7 (3–10)	8.55 ± 5.27	8 (4–12)	11.31 ± 7.79	9 (6–14.25) ^a^	14.25 ± 6.55	13 (9–18) ^a^	9.59 ± 6.54	8 (4–13)	<0.001 *
	**n**	**%**	**n**	**%**	**n**	**%**	**n**	**%**	**n**	**%**	***p*2**
**Female**	18	30.5	51	54.8 ^a^	52	70.3 ^a^	18	75.0 ^a^	139	55.6	<0.001
**Male**	41	69.5	42	45.2	22	29.7	6	25	111	44.4	

*p*1: One-way ANOVA × Kruskal–Wallis test, *p*2: chi-square test (^a^: normal), *: statistically significant values, SD: Standard deviation, IQR: Interquartile range, BMI: Body mass index.

**Table 2:** Beck score evaluations according to the HbA1c, fasting, and postprandial glucose levels.

	Beck Score								Total		
	Normal		Mild		Moderate		Severe				
	Mean ± SD (Min–Max)	Median (IQR)	Mean ± SD (Min–Max)	Median (IQR)	Mean ± SD (Min–Max)	Median [IQR]	Mean ± SD (Min–Max)	Median [IQR]	Mean ± SD (Min–Max)	Median (IQR)	*p*
**HbA1c**	7.16 ± 1.25 (5.1–11.2)	6.8 (6.2–7.7)	7.54 ± 1.63 (5.5–13.1)	7 (6.4–7.9)	9.1 ± 2.34 (5.4–15.9)	8.5 (7.48–10.63) ^ab^	9.98 ± 2.5 (5.9–15.3)	10.15 (7.98–11.95) ^ab^	8.15 ± 2.12 (5.1–15.9)	7.5 (6.5–9.2)	<0.001 *
**Fasting glucose**	139.92 ± 47.21 (77–285)	127 (108–164)	149.94 ± 51.61 (61–314)	138 (117–176)	210.14 ± 95.34 (75–495)	185 (131.75–266.25) ^a^	220.33 ± 117.74 (76–512)	202.5 (117–293.25) ^ab^	172.15 ± 80.82 (61–512)	142.5 (117–209.25)	<0.001 *
**Postprandial glucose**	188.2 ± 67.64 (101–405)	169 (134–230)	197.11 ± 75.36 (64–466)	176 (148–247.5)	263.86 ± 111.2 (98–612)	260.5 (172–304) ^ab^	272.63 ± 94.92 (95–405)	282.5 (194.5–366.5) ^ab^	222.02 ± 94.29 (64–612)	193 (149–282.25)	<0.001 *

*p*: Kruskal–Wallis test (^a^: normal, ^b^: mild), HbA1c: Glycated hemoglobin, *: statistically significant values, SD: Standard deviation, IQR: Interquartile range.

**Table 3:** Beck score evaluations according to the levels of HbA1c.

		Beck Score								Total		
		Normal		Mild		Moderate		Severe				
		n	%	n	%	n	%	n	%	n	%	*p*
**HbA1c**	<7	34	57.6	40	43	11	14.9 ^ab^	3	12.5 ^ab^	88	35.2	<0.001 *
	7–9	20	33.9	38	40.9	33	44.6	6	25	97	38.8	
	>9	5	8.5	15	16.1	30	40.5 ^ab^	15	62.5 ^ab^	65	26	

*p*: Chi-square test (^a^: normal, ^b^: mild), *: statistically significant value, HbA1c: Glycated hemoglobin.

**Table 4:** Beck score evaluations according to the antidiabetic agents.

		Beck Score								Total		
		Normal		Mild		Moderate		Severe				
		n	%	n	%	n	%	n	%	n	%	*p*
**Metformin**	Yes	35	59.3	54	58.1	27	36.5 ^ab^	10	41.7	126	50.4	0.015 *
	No	24	40.7	39	41.9	47	63.5	14	58.3	124	49.6	
**Sulfonylurea**	Yes	12	20.3	16	17.2	7	9.5	3	12.5	38	15.2	0.317
	No	47	79.7	77	82.8	67	90.5	21	87.5	212	84.8	
**SGLT-2-I**	Yes	13	22	17	18.3	20	27	4	16.7	54	21.6	0.523
	No	46	78	76	81.7	54	73	20	83.3	196	78.4	
**DPP-4 Inhibitor**	Yes	12	20.3	17	18.3	14	18.9	3	12.5	46	18.4	0.869
	No	47	79.7	76	81.7	60	81.1	21	87.5	204	81.6	
**DPP-4-I + Metformin**	Yes	15	25.4	20	21.5	22	29.7	2	8.3	59	23.6	0.173
	No	44	74.6	73	78.5	52	70.3	22	91.7	191	76.4	
**Glinides**	Yes	1	1.7	2	2.2	0	0	0	0	3	1.2	0.568
	No	58	98.3	91	97.8	74	100	24	100	247	98.8	
**Mixed Insulin**	Yes	1	1.7	5	5.4	13	17.6 ^ab^	2	8.3 ^ab^	21	8.4	0.005 *
	No	58	98.3	88	94.6	61	82.4	22	91.7	229	91.6	
**Basal Insulin**	Yes	2	3.4	7	7.5	11	14.9	2	8.3	22	8.8	0.125
	No	57	96.6	86	92.5	63	85.1	22	91.7	228	91.2	
**Intensive Insulin (Basal + 3 Bolus)**	Yes	8	13.6	8	8.6	26	35.1 ^ab^	15	62.5 ^abc^	57	22.8	<0.001 *
	No	51	86.4	85	91.4	48	64.9	9	37.5	193	77.2	
**Tiazolidines**	Yes	1	1.7	2	2.2	2	2.7	1	4.2	6	2.4	0.919
	No	58	98.3	91	97.8	72	97.3	23	95.8	244	97.6	
**Pioglitasone + Metformin**	Yes	1	1.7	4	4.3	0	0	0	0	5	2	0.209
	No	58	98.3	89	95.7	74	100	24	100	245	98	
**GLP-1 Analogue**	Yes	0	0	1	1.1	3	4.1	1	4.2	5	2	0.287
	No	59	100	92	98.9	71	95.9	23	95.8	245	98	
**Acarbose**	Yes	2	3.4	0	0	0	0	0	0	2	0.8	0.089
	No	57	96.6	93	100	74	100	24	100	248	99.2	

*p*: Chi-square test (^a^: normal, ^b^: mild, ^c^: moderate), *: statistically significant value, SGLT-2-I: Sodium–glucose cotransporter 2-1, DPP-4: Dipeptidyl peptidase-4, GLP-1: glucagon-like peptide 1.

**Table 5:** Logistic regression analysis and potential confounder parameters.

	Unstandardized Coefficients		Standardized Coefficients	95.0% Confidence Interval for B		*t*	*p*
	B	Std. Error	Beta	Lower Bound	Upper Bound		
**Constant**	−16.80	4.23		−25.13	−8.47	−3.972	**<0.001**
**Gender (male)**	4.14	0.81	0.24	2.55	5.73	5.120	**<0.001**
**Age**	0.24	0.04	0.29	0.16	0.31	6.206	**<0.001**
**Postprandial blood glucose**	0.02	0.01	0.17	0.00	0.03	2.467	**0.014**
**BMI**	0.23	0.09	0.12	0.05	0.42	2.497	**0.013**
**Mixed insulin**	5.27	1.45	0.17	2.41	8.13	3.635	**<0.001**
**Basal insulin**	5.85	1.38	0.19	3.13	8.57	4.236	**<0.001**
**Intensive insulin, Basal + Bolus, 4 Times/day**	8.73	0.98	0.43	6.80	10.66	8.905	**<0.001**
**HbA1c > 9**	2.63	1.43	0.13	−0.19	5.45	1.837	0.067

**Table 6:** Multiple linear regression analysis of various variables.

	Unstandardized Coefficients		Standardized Coefficients	95.0% Confidence Interval for B		*t*	*p*
	B	Std. Error	Beta	Lower Bound	Upper Bound		
**Constant**	−21.81	3.85		−29.39	−14.23	−5.667	**<0.001**
**Gender (female)**	4.23	0.80	0.25	2.66	5.80	5.303	**<0.001**
**Age**	0.23	0.04	0.28	0.16	0.30	6.248	**<0.001**
**BMI**	0.23	0.09	0.12	0.05	0.41	2.487	0.014
**Mixed insulin**	5.08	1.43	0.16	2.27	7.89	3.564	**<0.001**
**Basal insulin**	5.44	1.37	0.18	2.74	8.15	3.966	**<0.001**
**Intensive insulin, Basal + Bolus, 4 Times/day**	7.95	1.00	0.39	5.98	9.92	7.955	**<0.001**
**HbA1c**	1.50	0.19	0.37	1.12	1.87	7.784	**<0.001**

## Data Availability

The data that support the findings of this study are available on request from the corresponding author. The data are not publicly available due to privacy or ethical restrictions.

## References

[1] Roy T., Lloyd C.E. (2012). Epidemiology of depression and diabetes: A systematic review. J. Affect. Disord..

[2] Bădescu S.V., Tătaru C., Kobylinska L., Georgescu E.L., Zahiu D.M., Zăgrean A.M., Zăgrean L. (2016). The association between Diabetes mellitus and Depression. J. Med. Life.

[3] Moulton C.D., Pickup J.C., Ismail K. (2015). The link between depression and diabetes: The search for shared mechanisms. Lancet Diabetes Endocrinol..

[4] Vahia V.N. (2013). Diagnostic and statistical manual of mental disorders 5: A quick glance. Indian J. Psychiatry.

[5] Baumeister H., Hutter N., Bengel J., Härter M. (2011). Quality of life in medically ill persons with comorbid mental disorders: A systematic review and meta-analysis. Psychother. Psychosom..

[6] Chrousos G.P. (2009). Stress and disorders of the stress system. Nat. Rev. Endocrinol..

[7] Raison C.L., Capuron L., Miller A.H. (2006). Cytokines sing the blues: Inflammation and the pathogenesis of depression. Trends Immunol..

[8] Gonzalez J.S., Peyrot M., McCarl L.A., Collins E.M., Serpa L., Mimiaga M.J., Safren S.A. (2008). Depression and diabetes treatment nonadherence: A meta-analysis. Diabetes Care.

[9] DeFronzo R.A., Ferrannini E., Groop L., Henry R.R., Herman W.H., Holst J.J., Hu F.B., Kahn C.R., Raz I., Shulman G.I. (2015). Type 2 diabetes mellitus. Nat. Rev. Dis. Primers.

[10] Brieler J., Salas J., Scherrer J. (2022). Achievement of glycemic control and antidepressant medication use in comorbid depression and type II diabetes. Ann. Fam. Med..

[11] Association A.D. (2010). Diagnosis and Classification of Diabetes Mellitus. Diabetes Care.

[12] Elben S., Dimenshteyn K., Trenado C., Folkerts A.K., Ophey A., Sulzer P., Becker S., Schmidt N., Tödt I., Witt K. (2021). Screen Fast, Screen Faster: A Pilot Study to Screen for Depressive Symptoms Using the Beck Depression Inventory Fast Screen in Parkinson’s Disease With Mild Cognitive Impairment. Front. Neurol..

[13] Kant R., Yadav P., Barnwal S., Dhiman V., Abraham B., Gawande K. (2021). Prevalence and predictors of depression in type 2 diabetes mellitus. J. Educ. Health Promot..

[14] Albai O., Timar B., Braha A., Timar R. (2024). Predictive Factors of Anxiety and Depression in Patients with Type 2 Diabetes Mellitus. J. Clin. Med..

[15] Semenkovich K., Brown M.E., Svrakic D.M., Lustman P.J. (2015). Depression in type 2 diabetes mellitus: Prevalence, impact, and treatment. Drugs.

[16] Hoffman R.P., Damilano C.P., Hong K.M.C., Glick B.A., Kamboj M.K. (2022). Glycemic control, depression, diabetes distress among adolescents with type 2 diabetes: Effects of sex, race, insurance, and obesity. Acta Diabetol..

[17] Ali S.Y., Seid A.M., Hassen K., Abebe S.T., Banjaw Z., Ibrahim M. (2023). Depression and glycaemic control among adult patients with type 2 diabetes: A cross-sectional study in a comprehensive specialised hospital, Jigjiga, Ethiopia. BMJ Open.

[18] Lin K.D., Chang L.H., Wu Y.R., Hsu W.H., Kuo C.H., Tsai J.R., Yu M.L., Su W.S., Lin I.M. (2022). Association of depression and parasympathetic activation with glycemic control in type 2 diabetes mellitus. J. Diabetes Complicat..

[19] (1998). Intensive blood-glucose control with sulphonylureas or insulin compared with conventional treatment and risk of complications in patients with type 2 diabetes (UKPDS 33). UK Prospective Diabetes Study (UKPDS) Group. Lancet.

[20] Berge L.I., Riise T. (2015). Comorbidity between Type 2 Diabetes and Depression in the Adult Population: Directions of the Association and Its Possible Pathophysiological Mechanisms. Int. J. Endocrinol..

[21] Papelbaum M., Moreira R.O., Coutinho W., Kupfer R., Zagury L., Freitas S. (2011). glycemic control and type 2 diabetes. Diabetol. Metab. Syndr..

[22] Anderson R.J., Freedland K.E., Clouse R.E., Lustman P.J. (2001). The prevalence of comorbid depression in adults with diabetes: A meta-analysis. Diabetes Care.

[23] Guo M., Mi J., Jiang Q.M., Xu J.-M., Tang Y.-Y., Tian G., Wang B. (2014). Metformin may produce antidepressant effects through improvement of cognitive function among depressed patients with diabetes mellitus. Clin. Exp. Pharmacol. Physiol..

[24] Kessing L.V., Rytgaard H.C., Ekstrøm C.T., Knop F.K., Berk M., Gerds T.A. (2020). Antidiabetes Agents and Incident Depression: A Nationwide Population-Based Study. Diabetes Care.

[25] Perry B.I., Khandaker G.M., Marwaha S., Thompson A., Zammit S., Singh S.P., Upthegrove R. (2020). Insulin resistance and obesity, and their association with depression in relatively young people: Findings from a large UK birth cohort. Psychol. Med..

[26] Giannarelli R., Aragona M., Coppelli A., Del Prato S. (2003). Reducing insulin resistance with metformin: The evidence today. Diabetes Metab..

[27] Berge L.I., Riise T., Tell G.S., Iversen M.M., Østbye T., Lund A., Knudsen A.K. (2015). Depression in persons with diabetes by age and antidiabetic treatment: A cross-sectional analysis with data from the Hordaland Health Study. PLoS ONE.

[28] Soliman E., Essmat N., Mahmoud M.F., Mahmoud A.A.A. (2020). Impact of some oral hypoglycemic agents on type 2 diabetes-associated depression and reserpine-induced depression in rats: The role of brain oxidative stress and inflammation. Naunyn-Schmiedeberg’s Arch. Pharmacol..

[29] Woo Y.S., Lim H.K., Wang S.M., Bahk W.M. (2020). Clinical Evidence of Antidepressant Effects of Insulin and Anti-Hyperglycemic Agents and Implications for the Pathophysiology of Depression-A Literature Review. Int. J. Mol. Sci..

[30] Cha D.S., Best M.W., Bowie C.R., Gallaugher L.A., Woldeyohannes H.O., Soczynska J.K., Lewis G., MacQueen G., Sahakian B.J., Kennedy S.H. (2017). A randomized, double-blind, placebo-controlled, crossover trial evaluating the effect of intranasal insulin on cognition and mood in individuals with treatment-resistant major depressive disorder. J. Affect. Disord..

[31] Rotte M., Baerecke C., Pottag G., Klose S., Kanneberg E., Heinze H.J., Lehnert H. (2005). Insulin affects the neuronal response in the medial temporal lobe in humans. Neuroendocrinology.

[32] Grillo C.A., Piroli G.G., Kaigler K.F., Wilson S.P., Wilson M.A., Reagan L.P. (2011). Downregulation of hypothalamic insulin receptor expression elicits depressive-like behaviors in rats. Behav. Brain Res..

[33] Cai W., Xue C., Sakaguchi M., Konishi M., Shirazian A., Ferris H.A., Li M.E., Yu R., Kleinridders A., Pothos E.N. (2018). Insulin regulates astrocyte gliotransmission and modulates behavior. J. Clin. Investig..

[34] Shemesh E., Rudich A., Harman-Boehm I., Cukierman-Yaffe T. (2012). Effect of intranasal insulin on cognitive function: A systematic review. J. Clin. Endocrinol. Metab..

[35] Marks D.R., Tucker K., Cavallin M.A., Mast T.G., Fadool D.A. (2009). Awake intranasal insulin delivery modifies protein complexes and alters memory, anxiety, and olfactory behaviors. J. Neurosci..

[36] Zou X.H., Sun L.H., Yang W., Li B.J., Cui R.J. (2020). Potential role of insulin on the pathogenesis of depression. Cell Prolif..

[37] Bell R.A., Smith S.L., Arcury T.A., Snively B.M., Stafford J.M., Quandt S.A. (2005). Prevalence and correlates of depressive symptoms among rural older African Americans, Native Americans, and whites with diabetes. Diabetes Care.

[38] Noh J.H., Park J.K., Lee H.J., Kwon S.K., Lee S.H., Park J.H., Ko K.S., Rhee B.D., Lim K.H., Kim D.J. (2005). Depressive symptoms of type 2 diabetics treated with insulin compared to diabetics taking oral anti-diabetic drugs: A Korean study. Diabetes Res. Clin. Pract..

[39] Al-Amer R.M., Sobeh M.M., Zayed A.A., Al-Domi H.A. (2011). Depression among adults with diabetes in Jordan: Risk factors and relationship to blood sugar control. J. Diabetes Complicat..

[40] Bai X., Liu Z., Li Z., Yan D. (2018). The association between insulin therapy and depression in patients with type 2 diabetes mellitus: A meta-analysis. BMJ Open.

[41] Lackovic M.M., Macvanin M.T., Obradovic M.M., Gluvic Z.M., Sudar-Milovanovic E.M., Grujicic S.S., Isenovic E.R. (2023). Impact of treatment modalities on quality of life and depression in type 2 diabetes. Eur. Rev. Med. Pharmacol. Sci..

